# Distinct New York City *Aedes albopictus* Mosquito Populations Display Differences in Salivary Gland Protein D7 Diversity and Chikungunya Virus Replication

**DOI:** 10.3390/v12070698

**Published:** 2020-06-28

**Authors:** Maria E. Kaczmarek, Nora L. Herzog, Maria G. Noval, John Zuzworsky, Zahir Shah, Waheed I. Bajwa, Kenneth A. Stapleford

**Affiliations:** 1 Department of Microbiology, New York University Grossman School of Medicine, New York, NY 10016, USA; Maria.Kaczmarek@nyulangone.org (M.E.K.); Nora.Herzog@nyulangone.org (N.L.H.); maria.noval@nyulangone.org (M.G.N.); 2 New York City Department of Health & Mental Hygiene, New York, NY 10013, USA; jzuzwors@health.nyc.gov (J.Z.); zshah@health.nyc.gov (Z.S.); wbajwa@health.nyc.gov (W.I.B.)

**Keywords:** arbovirus, chikungunya virus, transmission, New York City, *Aedes albopictus*, saliva

## Abstract

In an increasingly interconnected world, the exposure and subsequent spread of emergent viruses has become inevitable. This is particularly true for *Aedes* (*Ae.*) mosquito-vectored viruses, whose range has increased over the past decade from tropical to temperate regions. However, it is unclear if all populations of *Ae.* mosquitoes in temperate New York City are able to successfully replicate and transmit arboviruses. To answer this question, we reared *Ae. albopictus* mosquitoes living in a temperate climate from three locations in New York City. We first sequenced the salivary antiviral protein D7 from individual mosquitoes in each population and found single nucleotide variants that are both shared and unique for each *Ae. albopictus* population. We then fed each population chikungunya virus (CHIKV) via an artificial blood meal. All three mosquito populations could be infected with CHIKV, yet viral titers differed between populations at 7 days post infection. Moreover, we found that these mosquitoes could transmit CHIKV to mice, and that virus RNA reached the saliva as early as two days post infection. Upon sequencing of the saliva CHIKV genomic RNA, we found mutations at sites correlated with increased transmission and virulence. These studies show that NYC *Ae. albopictus* populations can be infected with and transmit CHIKV, CHIKV is able to evolve in these mosquitoes, and that host salivary factors display population-specific diversity. Taken together, these studies highlight the need to study how distinct mosquito populations control viral infections, both at the virus and host level.

## 1. Introduction

Chikungunya virus (CHIKV) is an arthropod-borne virus primarily transmitted by the peridomestic mosquito, *Aedes (Ae.) aegypti* [1]. It causes a febrile illness accompanied by arthralgia of the joints, with occasional chronic arthralgia after virus clearance [2]. Unfortunately, there is no vaccine against CHIKV, leaving naïve human populations at risk of an epidemic [2]. Indeed, a recent outbreak in the Caribbean and the Americas resulted in 2.6 million confirmed cases, causing significant strain to both the healthcare system and economy in affected countries [3,4]. Due to the nature of CHIKV’s error-prone RNA-dependent RNA polymerase (2–4 mutations/10^4^ nucleotides in vivo and 600–1300 mutations/10^4^ nucleotides in vitro [5,6]) CHIKV has a high mutation rate, enabling it to sample many genotypes and adapt to novel environments. A prime example of CHIKV’s adaptability is the 2005 outbreak on La Réunion Island where over 300,000 people fell ill [4]. The success of this particular CHIKV strain was pinpointed to a single amino acid substitution that allowed for more efficient transmission by *Ae. albopictus*, yet did not affect transmission by *Ae. aegypti*, the original primary vector [7,8,9].

As CHIKV continues to extend beyond its endemic region, it is particularly disconcerting that certain CHIKV strains are transmitted by both tropical *Ae. aegypti* and temperate-tolerant *Ae. albopictus*. This expands the potential range of CHIKV beyond the equatorial region. Accordingly, a number of studies have been published attempting to characterize the vector competence of *Ae. aegypti* and *Ae. albopictus* mosquitoes in regions at risk of a CHIKV introduction [10,11,12,13,14,15,16,17]. Most populations of *Ae. albopictus* and *Ae. aegypti* tested from Europe, South America, and the United States (USA) have readily detectable CHIKV virions in saliva after feeding on an infectious blood meal. However, there exist both species level (between *Ae. aegypti* and *Ae. albopictus*) and population level differences for overall infectability, as well as degree and ease of dissemination throughout the mosquito body to the salivary gland [10,11,12,14,15,16]. Remarkably, even *Aedes* populations within the same city can differ in their transmission efficiencies of CHIKV [11], suggesting differences in either vector genetics or ecology across relatively short geographical distances.

The primary goal of the aforementioned studies is to describe the risk of transmission to humans. The metrics used are transmission rate and efficiency, which are a culmination of the virus’ traversal through the mosquito body. Both the midgut and the salivary gland present barriers the virus must overcome to successfully enter the saliva for transmission to mammals [18,19]. These barriers contain numerous obstacles (e.g., antiviral proteins, bacteria) that contribute to their ability to prevent or promote infection, which may differ between *Aedes* populations. For example, a recent predictive model based on empirical data shows that the number of viral particles needed for successful dissemination and eventual transmission varies greatly between mosquito populations found in Europe and China [10]. This would result in significant differences in ease of viral transmission in one region of the world versus another. One study focusing specifically on the salivary gland found differences at the level of the salivary gland exit barrier between *Ae. aegypti* and different U.S.A populations of *Ae. albopictus* [14]. Interestingly, for certain CHIKV strains where the salivary gland exit barrier was greatest in *Ae. aegypti*, the opposite was found in *Ae. albopictus*, where the salivary exit was not as great a barrier [14]. Together, these studies are evidence that the midgut and salivary gland can act as potent blocks, and categorizing an entire species as competent for CHIKV is not necessarily accurate. However, the genetic, anatomical, and microbial differences between mosquito populations are not completely understood. Elucidating this gap in knowledge would significantly advance our understanding of how specific mosquito populations control viral infections.

Therefore, it is essential to test specific mosquito populations for competency to assess the potential ease of spread of arboviruses in a non-endemic area, as well as to probe differences between mosquito populations [20,21,22,23,24,25,26]. Because New York City is a hub through which many people travel, there is the possibility of disease spread from distant locations. Here, we set out to test whether *Ae. albopictus* populations in New York City are competent for CHIKV in a laboratory setting. We also probe if these populations exhibit differences in the D7 long form salivary protein, a host factor known to influence viral infections [27,28]. In addition, as evidenced by the La Réunion outbreak, if novel viral genotypes emerge that are potentially advantageous, this can create a foothold from which an epidemic can be initiated. It can be argued that observing which genotypes emerge and are potentially transmitted to human hosts is an important aspect of vector capacity. However, there are few studies that have surveyed the CHIKV genotypes found in mosquito saliva as part of competence characterization. Here, we extracted RNA from mosquito saliva and Sanger sequenced the structural region of CHIKV to determine which mutations arose during viral replication in the vector. We found that indeed mutants arose with known virulence phenotypes in the saliva of local New York City *Ae. albopictus*. This is highly relevant for predicting which viruses will emerge in a non-endemic region.

## 2. Materials and Methods

### 2.1. Cell Culture and Generation of Viral Stocks

BHK-21 cells (ATCC CCL-10) were maintained in Dulbecco’s Modified Eagle Media (DMEM; Corning, Corning, NY, USA) supplemented with 10% fetal bovine serum (FBS; Atlanta Biologicals, Minneapolis, MN, USA), 1% nonessential amino acids (NEAA; Corning), and 1% penicillin/streptomycin (P/S; Corning) at 37 °C with 5% CO_2_. Vero cells (ATCC CCL-81) were maintained in DMEM supplemented with 10% newborn calf serum (NBCS) and 1% P/S at 37 °C with 5% CO_2_. All cell lines were confirmed to be mycoplasma free (Lookout PCR Detection Kit; Sigma-Aldrich, St. Louis, MO, USA).

The chikungunya virus (CHIKV) strain 06-409 (AM258994) was rescued from the CHIKV infectious clone [5]. Briefly, 10 μg of plasmid encoding the entire CHIKV genome was linearized using the restriction enzyme NotI. The linearized product was phenol-chloroform extracted, ethanol precipitated, and used as the template for in vitro transcription (SP6 mMessage mMachine, Ambion, Austin, TX, USA). After in vitro transcription, these nucleic acids were extracted using phenol–chloroform followed by ethanol precipitation. Subsequently, 10 μg of purified RNA was mixed with 3.9 × 10^6^ BHK-21 cells in a 2 mm electroporation cuvette and electroporated using 1 pulse at 1.2 kV, 25 mF and infinite resistance. Cells were transferred into 6 mL of complete media (DMEM with 10% FBS and 1% NEAA) and placed in a T25 flask at 37 °C for 72 h. The passage “zero” (P0) virus was collected after 72 h and clarified via centrifugation at 1200× *g* for 5 min. This was then used to infect a monolayer of BHK-21 cells (MOI ~ 0.01), and virus containing supernatants were collected 24 h later to generate the P1 stocks used in this study. Virus stocks were aliquoted and frozen at −80 °C, and viral titers were determined by plaque assay as described below. 

### 2.2. Mosquitoes

**Figure 1:**
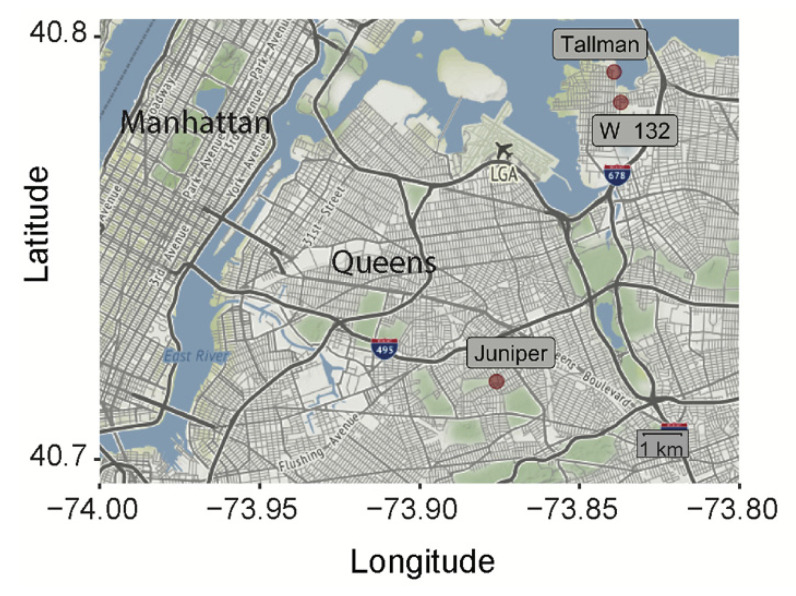
New York City map showing the exact geographical locations of *Ae. albopictus* collection sites (red dots).

*Ae. albopictus* eggs were collected from three locations in Queens, New York City (Figure 1) (132nd St, Whitestone, Queens: 40.78521, −73.83621; Juniper Valley Park, Middle Village, Queens: 40.72043, −73.87437; Powell’s Cove Blvd, College Point, Queens (Tallman Island Wastewater Treatment Plant): 40.792268, −73.83826) by the New York Department of Health.

*Ae. aegypti* (Poza Rica, Mexico; F20 plus) eggs were obtained from Dr. Gregory Ebel (Colorado State University) [29]. Mosquitoes were hatched and reared at 28 °C with 70% humidity and a 12 h diurnal light cycle in a climate controlled chamber (Memmert HPP750). *Ae. albopictus* mosquitoes used for infection did not exceed generation F8.

### 2.3. Mosquito Infections

Prior to infection with chikungunya virus, females from the three *Ae. albopictus* populations (Juniper, Tallman Island, and 132nd St, New York City, NY, USA) and *Ae. aegypti* (Poza Rica, Mexico) were sorted into pint cups and starved for 8 to 12 h. These mosquitoes were then exposed to an artificial blood meal containing between 1 and 4 × 10^6^ (high dose infection) infectious viral particles or containing 2 × 10^5^ and 7.5 × 10^5^ (low dose infection) infectious viral particles/mL diluted in PBS-washed sheep blood (Fisher Scientific, Waltham, MA, USA) supplemented with 5 mM ATP using a hemotek membrane feeding system for one hour. After feeding, engorged females were sorted into pint cups and incubated at 28 °C and 70% humidity with 10% sucrose ad libitum. Mosquitoes were collected and dissected at end time points—7 and 14 days post infection. At designated endpoints, mosquito legs and wings were removed and placed in a 2 mL roundbottom tube filled with 300 μL PBS and a 5 mm stainless steel ball (Qiagen, Germantown, MD, USA). Mosquitoes were then salivated by placing their proboscis into a 200 μL pipette tip filled with 5 μL of FBS. After one hour of salivation, the FBS was diluted in 45 μL of DMEM. The bodies were collected and placed in 300 μL of PBS in a 2 mL tube with a single 5 mm stainless steel ball. Legs and wings and bodies were homogenized with a Tissue Lyser II (Qiagen), and clarified by centrifugation at 8000 rpm for 8 min. Viral titers for saliva, bodies, and legs and wings were quantified by plaque assay (see below). Numbers of mosquitoes used in each replicate from each population can be found in Table S3 in the Supplementary Materials.

Infection rate was calculated as the total mosquitoes from which we detected virus using a plaque assay out of those that were engorged post feeding. Similarly, the dissemination rate was calculated as the total number of mosquitoes with detectable virus in the legs and wings of those mosquitoes that had virus-positive bodies. Finally, the transmission rate was calculated as the total number of mosquitoes with detectable viral particles in their saliva (using force salivation) out of those that had virus in their legs and wings.

### 2.4. Mouse Transmission Studies

Five to six week old male and female C57BL/6J were bred and reared in-house. Animal experiments were performed in accordance with all NYU School of Medicine Institutional Animal Care and Use Committee guidelines (IACUC). All mouse studies were performed using biosafety level 3 conditions. *Ae. albopictus* mosquitoes from Tallman (F7) were exposed to an artificial blood meal containing 1 × 10^6^ CHIKV infectious particles/mL [30]. Mosquitoes exposed to non-infectious bloodmeals were used as non-infected controls. After feeding, engorged females were sorted into pint cups and incubated at 28 °C and 70% humidity with 10% sucrose ad libitum. Seven and 11 days after the blood meal, mosquitoes were food-deprived for 12 h. Then, 5 to 6 week old C57BL6 mice were immobilized over a mesh covered pint cup containing the previously exposed to virus or exposed to blood only mosquitoes, and mosquitoes were allowed to feed for 40 min [31,32]. Each mouse was exposed to 1 to 5 mosquitoes. Afterwards, mice were returned to their cages, mosquitoes were killed and homogenized, and viral titers were determined by plaque assay. Mice were sacrificed at 2 and 3 days post transmission. Mice were euthanized by CO_2_ inhalation, and calf and quadriceps muscles were collected. Muscles were placed in a round-bottomed 2 mL tube containing 500 µL of PBS and two 5 mm stainless steel beads (Qiagen). Tissues were homogenized with a Tissue-Lyser II (Qiagen), and debris was pulled down through centrifugation at 8000 rpm for 8 min. Viral titers in tissue homogenates were determined by RT-qPCR.

### 2.5. Plaque Assay

Clarified virus containing supernatants were applied to a monolayer of Vero cells at 10-fold dilutions in order to determine plaque-forming units per milliliter (PFU/mL). Briefly, media was removed from cells, and virus diluted in DMEM was placed on the Vero monolayer for one hour at 37 °C and 5% CO_2_. Post incubation, cells were overlaid with 0.8% agarose in DMEM with 2% NBCS and incubated at 37 °C and 5% CO_2_ for 72 h. Cells were then fixed with 4% formalin, agarose plugs were removed, and wells were stained with crystal violet to quantify PFU/mL. Infectious titers were determined from the lowest dilution where plaques could confidently be counted.

### 2.6. RNA Extraction and RT-qPCR

RNA extractions were performed using TRIzol^TM^ reagent (Invitrogen, 250 μL of clarified tissue homogenate was added to 500 μL of TRIzol^TM^) following the manufacturer’s guidelines. The number of viral genomes/mL was quantified by RT-qPCR using the Taqman RNA-to-CT One-Step RT-PCR kit (Applied Biosystems^TM^, Beverly, MA, USA) and CHIKV primers to amplify a small region of nsp4 (primers in Table S1, Supplementary Materials) and a probe (5′-(6-carboxyfluorescein)-AGGTACGCGCTTCAAGTTCGGCG-(black-holequencher)-3′) targeting an amplicon in nonstructural protein 4 (nsP4) [5,31]. A standard curve was generated for each dataset using in vitro transcribed CHIKV RNAs.

### 2.7. Filter Paper Assay

Engorged female mosquitoes were individually housed in 50 mL conical tubes post blood feed [33]. Each individual mosquito was provided a 0.5 cm^2^ filter paper square soaked with 10% sucrose on which to feed. Filter papers were collected every 24 h for 4 days and placed directly into TRIzol^TM^ reagent. A new sucrose soaked filter paper square was provided each day. Filter papers were vortexed in TRIzol^TM^ reagent for approximately 30 s, and RNA was extracted following the manufacturer’s instructions and re-solubilized in water. This RNA was used as a template for cDNA synthesis (Maxima H Minus First Strand cDNA synthesis kit; Thermo Scientific, Waltham, MA, USA). cDNA served as a template for PCR (Phusion HF, Thermo Scientific) to amplify a 500 bp fragment of the 18S gene as well as a 500 bp fragment of the CHIKV E1 glycoprotein (primer sequences can be found in Table S1 in the Supplementary Materials). Individual PCR fragments were mixed with 6x DNA loading dye, separated on a 1% agarose-TAE (Tris, acetic acid, EDTA) gel, stained with ethidium bromide, and visualized on a Bio-Rad gel doc system.

### 2.8. Mosquito Saliva Sequencing

Mosquito saliva was collected as described above (Mosquito infection—force salivation). The DMEM plus saliva mixture was mixed with TRIzol^TM^ reagent, RNA extracted, and cDNA synthesized as described above (filter paper assay). We amplified the structural region of the CHIKV genome using two overlapping PCR products (Fragment 1 and Fragment 2—primer sequences found in Table S1, Supplementary Materials). Resulting PCR products of the correct size were purified using a PCR cleanup kit (Macherey–Nagel, Bethlehem, PA, USA) and Sanger sequenced (Genewiz, South Plainfield, NJ, USA) (sequencing primer sequences can be found in Table S1, Supplementary Materials).

### 2.9. D7 Sequencing from Mosquito Salivary Glands

Mosquito salivary glands were extracted from individual mosquitos and placed in TRIzol^TM^ reagent. RNA was extracted and used for cDNA synthesis as described above. The D7 long form transcript was amplified by PCR and Sanger sequenced (Genewiz) using the same primers (primer sequences can be found in Table S1 in the Supplementary Materials).

### 2.10. Virus and D7 Sequence Analysis

Virus Sanger sequencing results were aligned to the reference CHIKV sequence, strain 06-409. A mutation was considered real and significant if the peak of the non-WT nucleotide was at least half the amplitude of the original nucleotide. D7 Sanger sequencing results were aligned to the *Ae. albopictus* reference AALF024477 (VectorBase; www.vectorbase.org [34]. *Aedes albopictus* FOSHAN.AaloF1.2). A single nucleotide polymorphism (SNP) was called when the non-reference peak either replaced the reference peak or was approximately 50% of the reference peak (heterozygote).

### 2.11. Protein Structure Analysis

The CHIKV E1 protein (PDB: 3J2W) structure was analyzed using PyMOL (version 2.2.2).

### 2.12. Linear Modeling

The *lm* function in R (versions 3.3.3 and 3.6.3) was used to fit a linear regression to the mosquito infection data. Titers from body infection and saliva infection were all compared to one another to visualize and determine if a significant correlation existed. The *with* function was used to determine the direction of the correlation, i.e., negative or positive.

### 2.13. Data Availability

D7 sequences have been deposited in GenBank with accession numbers MT353980–MT354023.

### 2.14. Data analysis and Statistics

All statistical analysis and data visualization and editing were done in either GraphPad Prism (version 7.0b) or R Studio (version 1.2.5001). Agarose gels were analyzed using Image Lab and Photoshop. All experiments were completed in at least two independent biological replicates or using multiple individual mosquitoes. The specific statistical test and experimental N can be found in the figure legends. Tests for normal distribution (D’Agostino and Pearson, Shapiro–Wilk and Kolmogorov–Smirnov) were applied prior to choosing a statistical test to compare means. This influenced whether a parametric or non-parametric test was chosen.

## 3. Results

### 3.1. The D7 Long form Locus Reveals That NYC Ae. albopictus Populations Harbor Genetically Distinct Alleles

**Figure 2:**
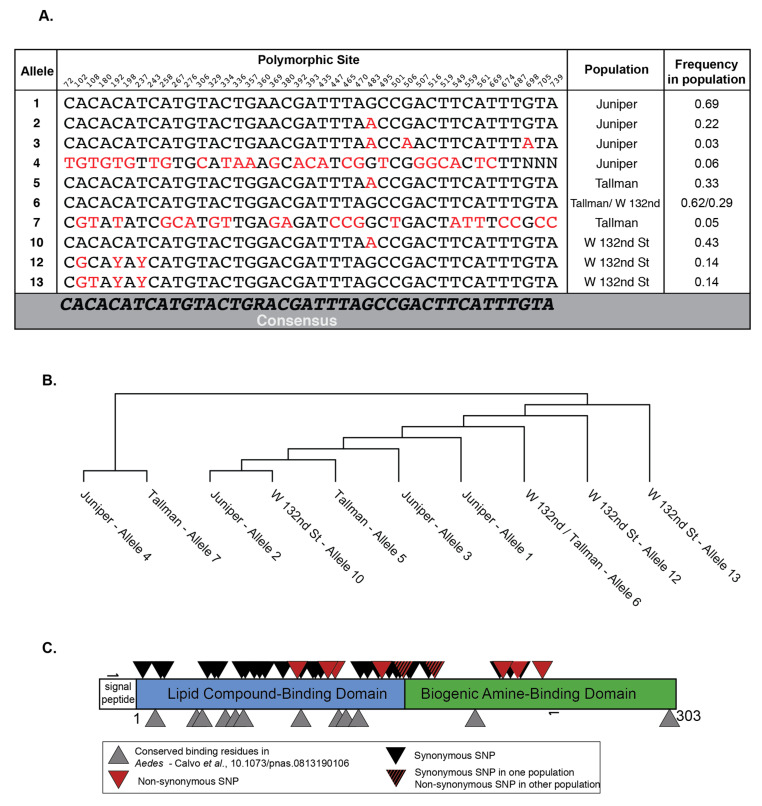
Individual mosquitoes and populations harbor unique D7 mRNA transcripts. (**A**) Table showing the nucleotides encoded at each polymorphic site of each allele found in all three populations (red). Table includes the allele’s frequency in each population in our samples. Numbers above each site represent the number of nucleotides in the D7 long form transcript based on reference sequence AALF02447 (VectorBase. www.vectorbase.org *Aedes albopictus* FOS-HAN.AaloF1.2). (**B**) Maximum parsimony tree showing relationship between alleles sampled in all populations. (**C**) Cartoon representation of the *Aedes* D7 long form showing the synonymous and non-synonymous variants found after Sanger sequencing the D7 long form transcripts isolated from individual salivary glands. Tallman *n* = 21, Juniper *n* = 16, W 132nd Street *n* = 7. The small arrows denote where primers used to amplify and sequence D7 long form lay.

Mosquitoes from three separate geographic locations were collected (Figure 1). The distances separating these locations are greater than 500 m, and therefore greater than the distance an *Ae. albopictus* mosquito is known to disperse [35,36,37]. Therefore, we were curious whether these populations may harbor genetically unique alleles. We focused on the D7 long form locus due to its known antiviral properties, and therefore functional relevance for viral transmission [27,28,29,30]. We Sanger-sequenced the D7 long form transcripts from individual mosquitoes from all three populations (Figure 2). The D7 family of long and short forms (not isoforms) is well characterized in blood feeding insects and has been previously described to have anti-pathogen functions [27,28,38,39]. Each individual’s alleles were phased based on assured alleles found in homozygous individuals. This inference was not always possible when there were multiple differences at unique sites, and the International Union of Pure and Applied Chemistry (IUPAC) ambiguity code is displayed (Figure 2A).

We observed 42 polymorphic sites in the D7 long form transcripts across all three populations (Figure 2). Of note, some of these sites were shared between populations, meaning that each population harbored individuals that had nucleotide differences at the same location in the D7 long form as the other populations (Figure 2A, red). Specifically, five sites with the same nucleotide polymorphisms were found in all three populations, while Juniper and Tallman shared an additional six sites (Figure 2A). For the most part, each population had its own unique alleles, found at intermediate frequencies, with only one allele shared between the Tallman and W 132nd St populations (Figure 2A and Figure S1). With further sampling, particularly of the 132nd St population, additional shared alleles may be found. The Tallman and W 132nd populations, which share an allele, are separated by a much shorter distance than Tallman or W 132nd street is from the Juniper population (Figure 1). It is therefore possible that populations separated by shorter distances have more gene flow (Tallman and W 132nd St.) versus those at greater distances from each other (Tallman or W 132nd Street and Juniper).

In order to visualize whether alleles were grouped by population, we analyzed the genetic relationship between alleles using a maximum parsimony tree (Figure 2B). Alleles found in an isolated population will have diverged from a founder genotype, linking them together in an evolutionary tree. We found that the two most divergent alleles, 7 and 4, lay outside the remainder of the major groupings (Figure 2B). This was unsurprising, as alleles 7 and 4 are rare and were separated by 23–25 sites from the most similar allele found in their respective populations (Figure S1B,C). The Juniper and W 132nd Street alleles were found to be mostly grouped by location. The exceptions to this statement were alleles 2 (Juniper) and 10 (W 132nd Street). These haplotypes we found to differ by a single mutation despite being from different populations (Figure 2B). The Tallman population harbored alleles that were dispersed throughout the tree (Figure 2B). A model where these populations were seeded from a single source and have now become isolated was supported by the lack of shared alleles found here (Figure 2A,C). This scenario would prevent recent gene flow, but allow for closely related and distinct genotypes in each population. However, multi-locus data from polymorphic sites of both nuclear and mitochondrial origin are necessary to definitively define population structures.

Due to the number of polymorphisms we observed in the D7 long form transcripts, we were curious whether any overlapped with known functional residues. Previous work has characterized the function of the protein domains found in the D7 long form, and comparative sequence analysis has identified conserved functional residues in mosquitoes [27]. Only one synonymous SNP overlapped with a known functional residue in the lipid compound-binding domain (Figure 2C). In addition, the SNPs resulting in non-synonymous changes did not overlap with known functional residues (Figure 2C). This was unsurprising as the housekeeping function of the protein must be maintained. Taken together, our results showed that mosquitoes from these three locations harbored genetically distinct D7 alleles.

### 3.2. NYC Ae. albopictus Mosquitoes from Different Locations are Competent for CHIKV Infection

**Figure 3:**
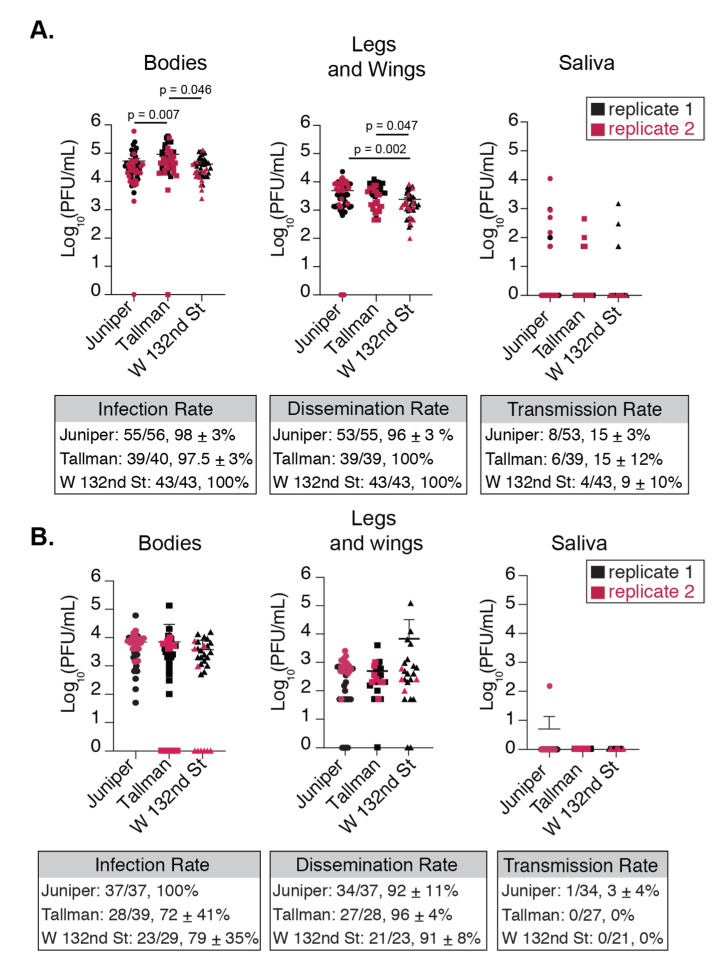
NYC *Ae. albopictus* populations are competent for CHIKV and display differences in virus replication. Titers of plaque forming units found in NYU *Ae. albopictus* bodies, legs and wings, and saliva at (**A**) 7 and (**B**) 14 days after infection with artificial bloodmeal containing 10^6^ PFU/mL of CHIKV IOL. Boxes below **A** and **B** show the infection, dissemination, and transmission rates which were calculated as the total number of mosquitoes with positive bodies, legs and wings, or saliva over the total number of engorged mosquitoes, positive bodies or legs and wings, respectively. Data represent two independent infections and graphs show the mean and standard deviation. Kruskall-Wallis with post-hoc Dunn’s Multiple Comparison test.

To determine whether NYC *Ae. albopictus* from distinct locations were similarly able to sustain a CHIKV infection, mosquitoes (generations F5–F8) were fed an artificial blood meal containing 10^6^ infectious viral particles per milliliter (PFU/mL) of CHIKV (Indian Ocean lineage). At 7 and 14 days post infection, mosquitoes were dissected and viral titers from bodies, legs and wings, and saliva were quantified by plaque assay (Figure 3A,B).

Here, we collected mosquito bodies, which constitute the carcass, salivary glands, and the primary site of infection—the midgut. We also collected legs and wings and saliva to determine how the virus disseminated from the main body cavity to extremities and out of the salivary gland. The numbers of mosquitoes for each replicate from each population can be found in Table S3 of the Supplementary Materials. Viral titers from bodies are indicative of virus passing into the midgut, the primary site of replication. At day 7 post infection, we found that overall infection rates, represented as the percentage of mosquitoes with detectable virus in their bodies, were nearly 100% and similar between *Ae. albopictus* populations (Figure 3A, Table S2). Interestingly, at day 7 we found significant differences in viral titers in the bodies between *Ae. albopictus* populations (Figure 3A); however, those differences disappeared at 14 days post infection (Figure 3B). While mosquitoes from all three locations are readily infected with CHIKV, the level of infection differs significantly between them.

Next, we determined to what degree CHIKV disseminated out of the midgut by quantifying the infectious particles in the legs and wings of each mosquito. Similar to the infection rate, at day 7 post infection we found that the dissemination rate was near 100% and consistent between populations (Figure 3A, Table S2). As we saw with the mosquito bodies, there existed population-specific differences in the leg and wing titers at 7 days post infection (Figure 3A), yet this again went away at 14 days post infection (Figure 3B). The population with the highest body titers, Tallman, did not exhibit the highest titers in the legs and wings at 7 days post infection. Additionally, *Ae. albopictus* from W 132nd St. harbored consistently lower concentrations of virions across their bodies and legs and wings at 7 days post infection. Together, these data suggest that CHIKV can establish infections in NYC *Ae. albopictus* mosquitoes, and that there exist population-specific barriers to the number of viral particles that disseminate.

**Figure 4:**
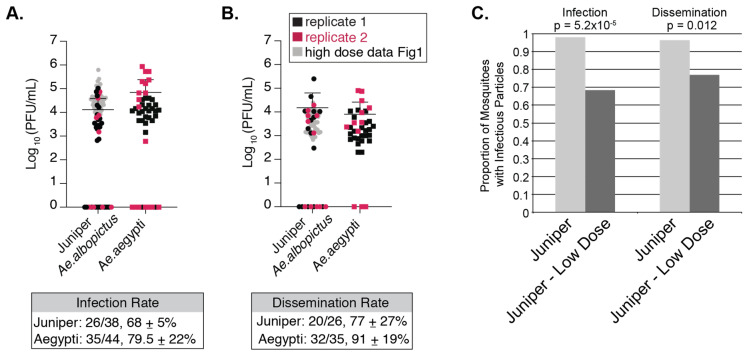
Lower dose infections of *Ae. albopictus* result in lower infection and dissemination rates. Titers of plaque forming units found in NYC *Ae. albopictus* bodies (**A**) and legs and wings (**B**) 7 days post infection with artificial bloodmeal containing 10^5^ PFU/mL of CHIKV IOL. The gray spheres are the results from the high dose (10^6^ PFU/mL) infection of Juniper *Ae. albopictus* mosquitoes. The box below graph A. represents the number of mosquitoes that were infected from those that bloodfed. The box below graph B. represents the total number of mosquitoes with infectious particles detected by plaque assay in legs and wings out of those with detectable infectious particles in their bodies. Standard deviation for both A. and B. was calculated using 2 replicates shown on the graphs above. (**C**) Frequencies of body (infection) and leg and wing (dissemination) infections from regular dose (10^6^ PFU/mL) and low dose infections (10^5^ PFU/mL) of Juniper mosquitoes. Fisher’s exact test was implemented to compare frequencies (infection *p*-value *p* < 0.001; dissemination *p*-value *p* < 0.05).

In nature, viremia of infected people varies widely, which may impact mosquito infection rates [40]. Therefore, we further probed NYC mosquito competence for CHIKV by feeding *Ae. albopictus* from the Juniper population and *Ae. aegypti* (Poza Rica, Mexico, control) a lower-dose of CHIKV (10^5^ PFU/mL of CHIKV). *Ae. aegypti* mosquito bodies had similar titers to NYC *Ae. albopictus* 7 days post infection (Figure 4). Titers were also consistently similar in the legs and wings (Figure 4). When comparing high and low dose infections, we found that Juniper *Ae. albopictus* that had taken the low dose bloodmeal had significantly lower infection rates, dissemination rates (Figure 3, Supplementary Materials Table S2), and lower CHIKV body titers compared to our high dose infection of mosquitoes from the same location (Figure 4—see overlap between gray and colored dots, Welch’s *t*-test, *p* < 0.05). However, there was no significant difference between low and high dose infection titers in legs and wings in Juniper *Ae. albopictus* (Welch’s *t*-test, *p* > 0.05, see overlap between gray and colored dots in Figure 4). This shows that low dose infections can impact overall infection and dissemination rates in mosquitoes, yet once the mosquito is infected, the virus is likely to reach similar titers in mosquito extremities.

### 3.3. NYC Ae. albopictus Mosquitoes Can Transmit CHIKV

The salivary gland, like the midgut, can pose as a physical and biochemical barrier to viral replication and therefore transmission. Hence, we used multiple approaches to assess the dynamics of transmission and whether NYC *Ae. albopictus* mosquitoes could transmit CHIKV. First, at 7 and 14 days post infection, we allowed mosquitoes to salivate into a pipet tip containing FBS as we have done before [31]. Using this method, the transmission rates were far lower than the rates of dissemination for all mosquito populations (Figure 3A—legs and wings versus saliva). For those mosquitoes where virus was detected in the saliva, Juniper titers trended higher compared to the other *Ae. albopictus* populations. However, at 14 days post infection almost no infectious particles were detected in the saliva from any of the *Ae. albopictus* populations (Figure 3B—Saliva), suggesting that the optimal time for transmission for these mosquitoes is early during infection.

Since we detected few mosquitoes with viral particles in their saliva, we investigated whether a higher viral load in mosquito bodies resulted in increased dissemination to the extremities and the salivary gland. Thus, we determined whether body titer was correlated to (i) leg and wing or (ii) saliva titer, as well as whether (iii) body titer was a significant predictor of leg and wing titer. These analyses were meant to inform whether a high body titer is predictive of dissemination to legs and wings or salivary glands. While we did find a positive correlation between the titers in mosquito bodies and legs and wings (Figure S2A), there was no significant relationship between body or leg and wing titers with saliva titers (Figure S2B,C).

**Figure 5:**
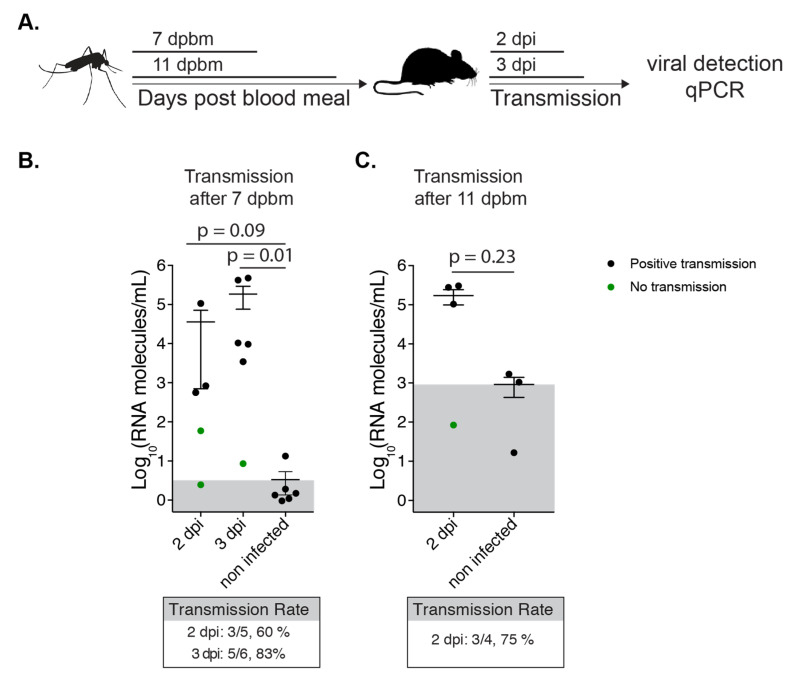
NYC Tallman mosquitoes can transmit CHIKV to mice. (**A**) Schematic representation of the experimental setup. (**B** and **C**). 7 day old Tallman *Ae. albopictus* were infected with 10^6^ PFU/mL CHIKV infectious bloodmeal. After 7 (**B**) or 11 (**C**) days post bloodmeal (dpbm) mosquitoes were allowed to feed on adult C57BL/6J mice. Viral RNA genomes extracted from calf and quadricep muscles were quantified by qPCR. Transmission rate was defined as the number of infected mice over the number of total mice. *p*-values were determined by Mann-Whitney test. Gray boxes indicate the position of the mean from the negative controls.

Ultimately, we were interested in the ability of NYC *Ae. albopictus* to transmit virus to a mammalian host. Thus, we allowed Tallman mosquitoes at 7 and 11 days post artificial infectious blood meal to feed on 5 to 6 week old C57BL6 mice (Figure 5A). Because CHIKV is a joint and muscle tropic virus, we determined the number of CHIKV genomes in the quadricep and calf muscle of the mice to assess transmission (Figure 5B,C) and quantified the number of infectious virus in each mosquito to assess initial infection (Figure S3). We observed that NYC *Ae. albopictus* mosquitoes could directly transmit CHIKV as early as 7 days and as late as 11 days post infection, as evidenced by the presence of RNA above background levels (Figure 5B,C). Additionally, we found the transmission rate to be ~60 to 80%. This is a much higher rate than was suggested when using a pipet tip for salivation of the Tallman population at 7 days (Figure 1—saliva; 15%). Altogether, NYC *Ae. albopictus* were able to transmit virus to mammals via bite, and a transmission model may be a more accurate assessment of transmission rate compared to pipet tip salivation.

**Figure 6:**
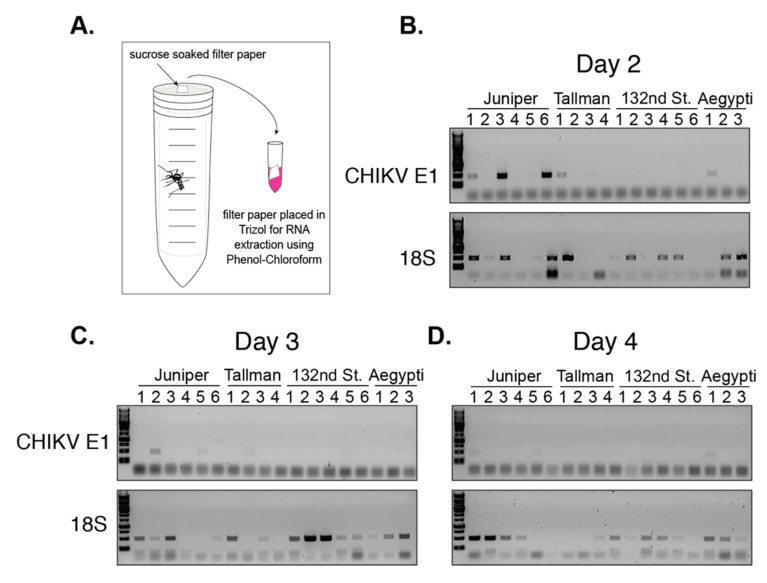
Viral genomic fragments can be detected in NYC *Ae. albopictus* saliva as early as 2 days post infection. (**A**) Schematic of experimental setup. CHIKV RNA was extracted from filter paper at 2 (**B**), 3 (**C**), and 4 (**D**) days post infection. Representative images of 1% agarose gels depict the results from amplification of a 500 bp 18S fragment and a 500 bp CHIKV E1 glycoprotein fragment. Each lane represents a single individual mosquito.

An important aspect of transmission efficiency and spread of an arbovirus is the extrinsic incubation period (EIP). EIP is a measure of how quickly viral particles reach the saliva of a mosquito. CHIKV is known to have a relatively short EIP; it takes from 2 to 5 days for infectious particles to reach saliva in *Aedes* species [41,42,43,44,45]. Thus, we hypothesized that transmission may occur early during infection in NYC *Ae. albopictus* mosquitoes. To begin to assess this, we determined whether individual mosquitoes had viral RNA in their saliva during the 4 day period post infectious blood meal [33]. This method does not directly quantify viral particles; however, it allows for the repeat sampling of the same individual mosquitoes over the course of 4 days. Briefly, mosquitoes were fed an artificial infectious blood meal, and individually housed mosquitoes were presented with sucrose soaked filter papers for feeding (Figure 6A).

Filter papers were collected every 24 h and placed in TRIzol^TM^ for RNA extraction (Figure 6A). For each time point we amplified a fragment of the CHIKV E1 glycoprotein. As a control, we amplified a fragment of mosquito ribosomal 18S RNA to determine whether the mosquito fed and whether we had abundant and high-quality RNA for amplification. *Ae. aegypti* (Poza Rica) was included as a control because CHIKV detection in saliva at 2 to 5 days is described for this species and it is the primary vector [14,19]. We were able to amplify 18S from at least one time point from nearly every individual (Figure 6—18S). In addition, viral RNA was detected in the saliva of at least one *Ae. aegypti*, and 18S was consistently amplified each day from nearly all of the *Ae. aegypti* mosquitoes (Figure 6). We observed that viral RNA reached the salivary gland by two days post infection in four *Ae. albopictus* individuals from Juniper and Tallman; yet, we were unable to detect any viral RNA in mosquitoes from the 132nd Street population (Figure 6). This is consistent with the 132nd Street population having the lowest transmission rate (Figure 3, Table S2). From these studies, we were able to establish that CHIKV RNA is detected in NYC *Ae. albopictus* saliva as early as two days post infection, suggesting that these mosquitoes can potentially transmit CHIKV early after initial infection.

### 3.4. Within-Host Evolution of CHIKV in NYC Ae. albopictus Reveals Previously Identified Transmission Variants

**Figure 7:**
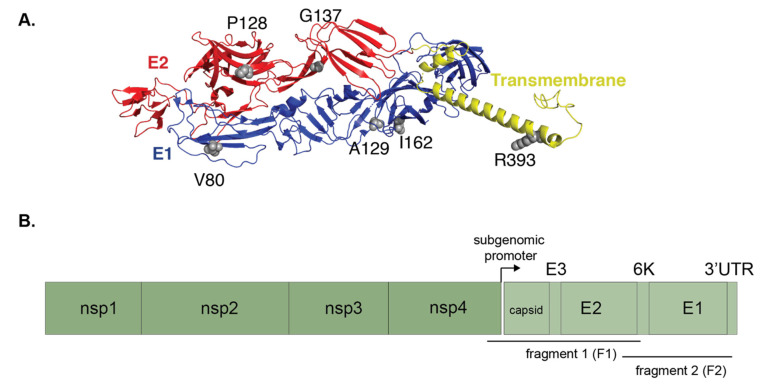
Substitutions accumulated by CHIKV during NYC *Ae. albopictus* infection are found at sites associated with adaptive evolution. (**A**) The crystal structure of the E1 (blue) and E2 (red) glycoproteins, along with the transmembrane domain (yellow) of CHIKV is shown (PDB: 3J2W). Any residue at which a mutation occurred is shown as gray spheres and labeled. (**B**) Domain diagram shows CHIKV genome and amplified fragments.

Previous CHIKV evolution experiments in *Ae. albopictus* from diverse geographical regions revealed the appearance of mutations in the E1 glycoprotein resulting in phenotypes that modulate transmission and virulence in mouse models [31]. We wanted to know if similar mutations arose during CHIKV replication in NYC *Ae. albopictus* mosquitoes. RNA was extracted from mosquito saliva, and the subgenomic region of the CHIKV genome was amplified in two fragments and Sanger sequenced (Figure 7B). From those mosquitoes where we successfully amplified the target region, four out of the seven mosquitoes contained variants at sites 80 and 129 of the E1 glycoprotein (Figure 7, Table 1). These sites are significant because they are linked to phenotypes resulting in increased virulence and transmission for CHIKV [30,31]. 

In particular, the pathogenic variant V80I was found in three mosquitoes. Two other amino acid substitutions at site 129 were found in two separate mosquitoes, E1 A129F and A129W (Figure 7B, Table 1). In mosquitoes that did not have either of these mutations (E1-80 and/or 129), we found novel synonymous mutations in E1 and E3 and nonsynonymous changes in the E2 or E3 structural proteins (Figure 7B). The mutations in CHIKV E1 (V80I, I162V, and A129W/F) were situated along the entirety of the E1 glycoprotein, with site I162 proximal to A129 (Figure 7A, sites in gray). These data suggest that CHIKV may sample similar mutations in multiple mosquitoes from around the world, and that selection of these variants may be due to a common environmental or host factor such as temperature or saliva microenvironment.

## 4. Discussion

Chikungunya virus (CHIKV) is an emerging virus, whose range has rapidly expanded over the past two decades. In 2013 the Asian lineage of CHIKV reached the Caribbean, where it had not previously sustained autochthonous transmission, and spread to Central and South America resulting in 2.6 million reported cases of disease [3,4,46]. CHIKV’s rapid dispersal necessitates a competent vector. Competence describes the ability of a vector to become infected, maintain, and potentially transmit the infectious agent [47,48]. In addition to competence, a number of other intrinsic (daily blood feeding rate, the extrinsic incubation period for the virus, and probability of survival [18]) and extrinsic (environmental [25] and microbial [49,50]) qualities affect the capacity of the vector to launch an epidemic. Previous work has shown that *Aedes* populations around the globe are relatively susceptible to numerous non-endemic arboviruses [47]. However, empirically derived threshold values describing the number of viral particles required for dissemination in mosquitoes have revealed differences between European and Asian mosquitoes, with some populations requiring a lot more virus particles for transmission [10]. In addition, some studies have shown that dynamics of infection, i.e., how quickly CHIKV appears in saliva across a 10 to 14 day period, varies [16,41,42,43,44,45]. Therefore, we set out to specifically study the degree of infection, infection rate, dissemination rate, and transmission rate of three populations of *Ae. albopictus* found in Queens, New York City, infected with the Indian Ocean Lineage strain of CHIKV.

To both characterize the salivary microenvironment as well as determine potential genetic divergence at an antiviral locus in these populations, we sequenced the D7 long form salivary messenger RNA transcript. We found that each population harbors unique genetic variants. Further sampling would be required to determine the absolute haplotype networks of these mosquito populations, and mechanistic validation is required to determine whether these variants function differentially with respect to viral inhibition. However, these initial studies highlight the genetic differences amongst individuals within and between populations of a single mosquito species residing in one city.

We found significant differences in CHIKV titers in both bodies and legs and wings between populations (Figure 3). These data support the hypothesis that these populations are likely distinct, and that there are unique obstacles to the number of disseminated viral particles in individuals from discrete locations. In general, we found that all three populations of *Ae. albopictus* isolated from Queens, New York City are susceptible to the Indian Ocean Lineage strain of CHIKV (Figure 3, Figure 4 and Figure 5). In addition, NYC *Ae. albopictus* continue to harbor virus 14 days after infection, in-line with previous work showing that *Aedes* species are able to sustain CHIKV replication for weeks after the onset of the infection [31,42]. Yet, unlike previous work where titers in bodies and legs and wings were maintained at 14 days, we find titers drop by a log after 14 days compared to 7 days post infection. This may suggest certain barriers to viral replication or an eventual clearing of virus over time, which is common to all three populations. It is possible that experimental setup influenced our results, leading to lower rates of infection and therefore dissemination. However, replicate infections were done at similar times of day, weeks apart, and with independently reared batches of mosquito eggs, and yet still resulted in overlapping titers (Figure 3). It should be noted that infection and dissemination rates remained stable and above 72% 14 days after infection (Figure 3), albeit a drop in titers. It is possible NYC *Ae. albopictus* are better able to control levels of infection, yet remain productively infected.

Importantly, for competence, virus must enter the saliva in order to be transmitted. We used a non-invasive filter paper method that allowed us to monitor viral RNA in individual mosquito saliva over a 4-day period. We observed transmission of viral RNA as early as two days post infection for two of the NYC *Ae. albopictus* populations, suggesting that these mosquitoes potentially transmit virus early after infection. In order to directly determine possible transmission rates, we used two methods: (i) salivation into a pipette tip (force salivation) and (ii) an in vivo transmission model. Using the pipette tip method, we calculated transmission rates between 3 and 10% for 7 days post infection, which while low, would still constitute a large number of mosquitoes given their population size (Figure 3). Importantly, we also evaluated transmission rates using an in vivo transmission model where infected mosquitoes were able to feed on mice. We found *Ae. albopictus* from Tallman island could transmit virus to mice at 7 and 11 days post infection, with a transmission rate ranging around 60 to 80% (Figure 5). This discrepancy between methods is likely technical due to the fact that (1) the pipette method does not allow for us to assess whether the mosquito has salivated into the pipette tip used for collection, and (2) mice were fed on by multiple mosquitoes. Additionally, each method is quantifying virus at different points in a transmission cycle. When using force salivation we are attempting to assess the initial inoculum that would be transmitted to a mammal. In the mouse transmission experiment, the initial inoculum is transmitted and the infected host tissue collected 2 or 3 days later, which assesses whether the virus successfully replicated in the host target organ. Taken together, these transmission rates are lower than those reported in studies elsewhere that used a force salivation method for saliva collection. Those studies report a range between 30% and 80% [11,14]. Accordingly, this may suggest NYC *Ae. albopictus* populations have a more robust salivary gland exit barrier or a saliva specific biochemical barrier. Finally, our data did not suggest an association between high body titers or leg and wing titers and presence of virus in the saliva (Figure S2). Again, this potentially supports a barrier to transmission at the level of the salivary gland. No specific molecules have been described as salivary gland exit barriers for CHIKV in *Ae. albopictus.* Studies of *Ae. aegypti* salivary transcripts have identified specific transcripts expressed in separate lobes of the salivary gland, potentially influencing where virus can replicate and is found [51,52]. Additionally, changes in protein expression occur in the salivary gland of *Ae. aegypti* post CHIKV infection, suggesting a role for specific proteins [53]. Further work is necessary to pinpoint which proteins influence viral infection, their mechanisms, and whether they impact viral selection.

Another important aspect of emerging virus dynamics is the potential to adapt to a new vector, or acquire novel traits that aid in transmission. Here, we find the emergence of unique viral variants in saliva after 7 days of replication in NYC mosquitoes (Figure 7, Table 1). Previous work characterizing adaptive mutations in the E1 glycoprotein has shown that mutations at sites V80 and A129 can result in increased virulence and transmission efficiency in mice [30,31]. These mutations arose during experimental evolution in the laboratory, similar to the setting in which we conducted this study. Interestingly, we find the same exact substitutions at site E1-80, a valine to an isoleucine, in mosquitoes from each population (Figure 7B). In addition, we find substitutions at site E1-129 and nearby at E1-162 (Figure 7A). This suggests that these substitutions arise in viral populations replicating in *Aedes* species regardless of location, potential differences in host genetics, and microbiota. Given that we sampled only a few mosquitoes, it is remarkable that these evolutionary events are recapitulated. And while virulent phenotypes have been associated with mutations at sites E1-80 and 129, it is still unknown why they emerge in saliva. Therefore, NYC *Ae. albopictus* can potentially serve as a model to study the mechanism driving the emergence of these CHIKV variants. Taken together, these studies emphasize the need to proactively study arbovirus infections in naïve mosquito populations. This allows us to better understand how distinct mosquito populations control arbovirus infections, and how arboviruses may evolve when introduced to a novel host during an epidemic.

## Figures and Tables

**Table 1:** Mutations that arise during the course of 7 day infection in NYC mosquitoes.

Mosquito #—Fragment Amplified	Location	Gene	Amino Acid	Codon
1—F1, F2	Juniper	E1	S25	Syn
		E1	V80I	GTC to ATC
		E1	A129F	GCA to TTT
		E1	A129F	GCA to TTT
		E1	A129F	GCA to TTT
		3’UTR	n/a	n/a
2—F1	Tallman	E3	T34	Syn
		E2	P128L	CCA to CTA
		E2	G137D	GGT to GAT
		E2	R393Q	CGA to CAA
3—F2	Juniper	E1	V80I	GTC to ATC
		E1	I162V	ATT to GTT
4—F2	W 132	E1	V80I	GTC to ATC
		E1	S111	Syn
5—F2	Tallman	E1	A129W	GCA to TGG
		E1	A129W	GCA to TGG
		E1	A129W	GCA to TGG

## Supplementary Materials

The following are available online at https://www.mdpi.com/1999-4915/12/7/698/s1, Figure S1: Haplotypes present in individual mosquito populations; Figure S2: Saliva titers are not correlated to body or legs and wings titers in NYC *Ae. albopictus*; Figure S3: Mosquitoes used in transmission experiment were infected with chikungunya virus; Table S1: Primers used in this study; Table S2: Statistical analysis of infection, dissemination, and transmission rates by Fisher’s exact test; Table S3: Number of mosquitoes used in each experiment.
