# Supplementary material for: Distinct New York City Aedes albopictus Mosquito Populations Display Differences in Salivary Gland Protein D7 Diversity and Chikungunya Virus Replication
Source: Viruses. 2020 Jun 28;12(7):698. doi: 10.3390/v12070698 (PMC7411853; doi:10.3390/v12070698)
Supplement: Supplementary file 1 [file viruses-12-00698-s001.pdf]

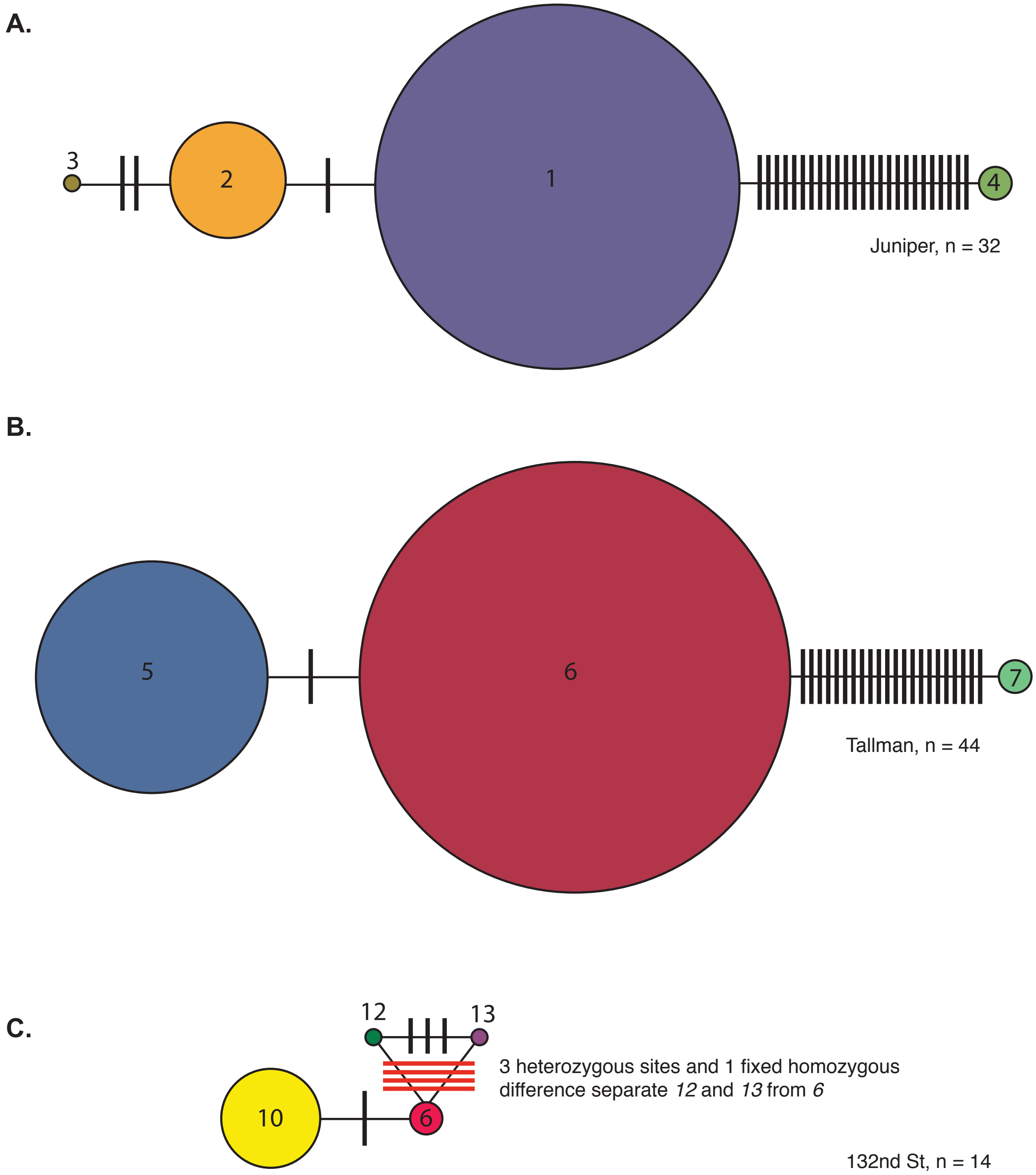

Figure S1. Haplotypes present in individual mosquito populations. Haplotype network of alleles found in (A) Juniper, (B) Tallman, and (C) W. 132nd street. Size of circles is representative of number of specific allele found in population. Small black and red bars indicate 1 nucleotide difference between alleles.

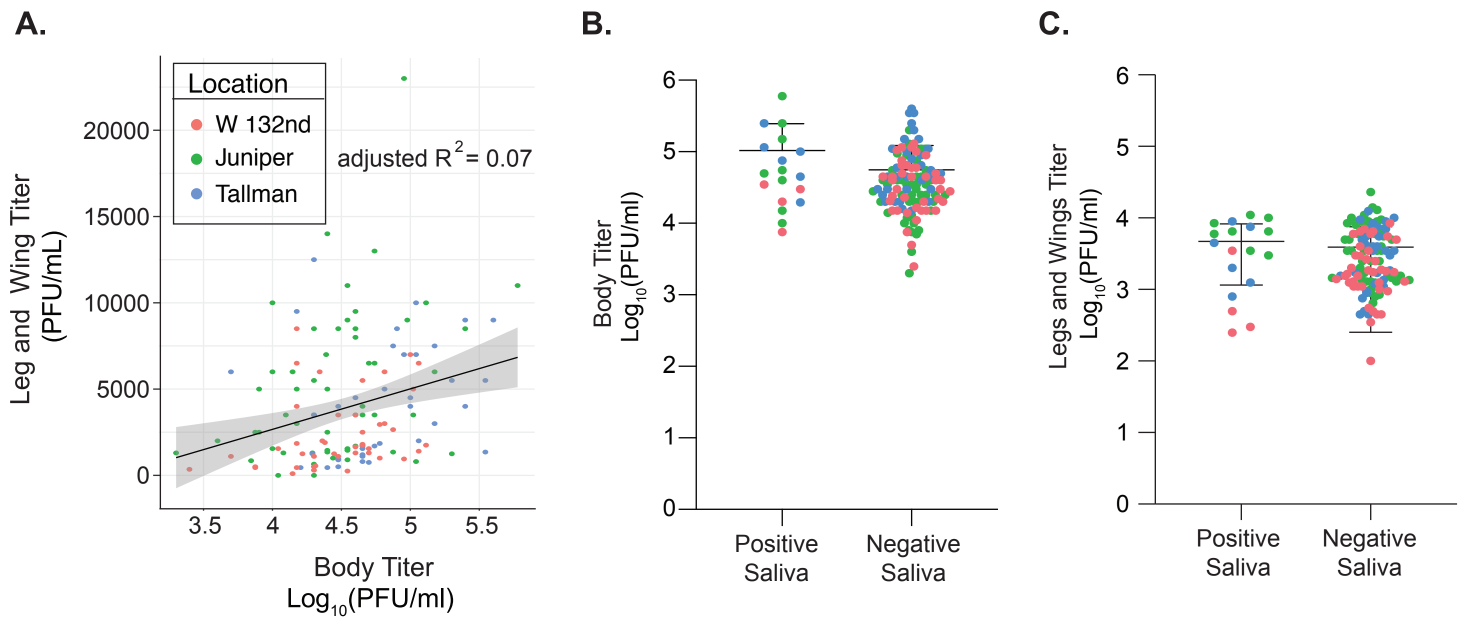

Supplemental Figure 2. Saliva titers are not correlated to body or leg and wing titers in NYC Ae.albopictus. (A) The linear correlation between body and leg and wing titers is shown (p-value <0.01). The gray area represents the 95 percent confidence interval from the linear model. (B) Body titers of mosquitoes that had infectious saliva is compared to those that did not (Welch's t-test, p>0.05). Similarly in (C) Leg and wing titers of mosquitoes that had infectious saliva is compared to those that did not (Welch's t-test, p>0.05).

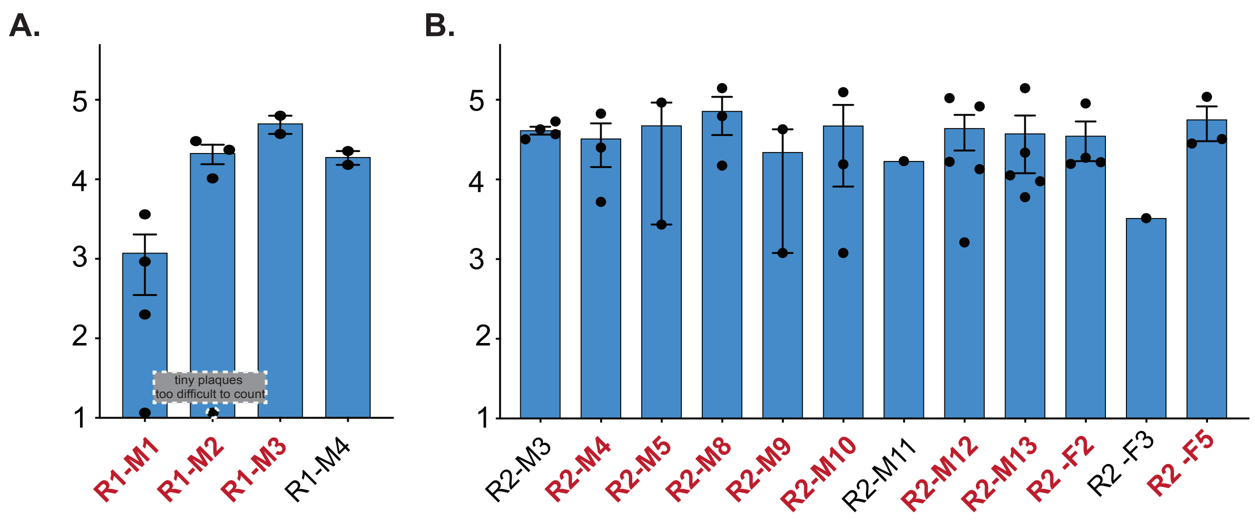

Supplemental Figure 3. Mosquitoes used in transmission experiment were infected with chikungunya virus. Mosquitoes were fed an artificial blood meal spiked with chikungunya virus, and engorged mosquitoes were sorted. 7 to 11 days post infection these mosquitoes were allowed to feed on mice. Each mouse was exposed to 1 to 5 mosquitoes. Each dot represents the titer in a single mosquito body determined by plaque assay after feeding on the mice. The mice highlighted in red were successfuly infected. (A) replicate 1 and (B) replicate 2.

Table S1: Primers used in this study

Method / Purpose	Forward (5' - 3')	Reverse (5' - 3')
qPCR/ quantify CHIKV genomes	TCACTCCCTGCTGGACTTGATAGA	TTGACGAACAGAGTTAGGAACATACC
PCR/ *Ae. albopictus* 18S fragment	GGTCGGCGCGGTCGTAGTGTGG	TCCTGGTGGTGCCCTTCGTCAAT
PCR/ CHIKV E1 fragment	CAACGAGCCGTATAAGTATTGG	CCCTTTGAACTACTTCTGT
PCR/ CHIKV structural region, fragment 1	TTAAACTGGGCAAACCGC	GCCTCTTGGTATGTGGCCGC
PCR/ CHIKV structural region, fragment 2	CAACGAGCCGTATAAGTATTGG	CCCTTTGAACTACTTCTGT
PCR and Sequencing/ *Ae. albopictus* D7 long form	CGGGACGTGAAGGAAAAATCG	TTGAGCCTGAACTTTTGCATC

Sequencing Primers for CHIKV E1	5' - 3'
**for fragment 1**	GAGGCCGGTTCACCATCC
	GTTTCCTCCGGTTCCTTTTC
	GAACGACAGACGGGACG
	CAACGAGCCGTATAAGTATTGG
**for fragment 2**	GAACGAGCAGCAACCTTTG
	TCTCCGTACGTGAAGTGCTG
	GCCCTCTTTAACGGACATG
	CATGGTCGCCGTTTGCATA

Table S2: Statistical analysis of infection, dissemination, and transmission rates by Fisher's exact test.

Populations Compared	High Dose - 7 day Infection		
	Infection Rate	Dissemination Rate	Transmission Rate
Juniper v. Tallman	not sig	not sig	not sig
Juniper v. 132nd St	not sig	not sig	not sig
Tallman v. 132nd St	not sig	not sig	not sig
**Populations Compared**	**High Dose - 14 day Infection**		
	Infection Rate	Dissemination Rate	Transmission Rate
Juniper v. Tallman	p = 0.001	not sig	not sig
Juniper v. 132nd St	p = 0.016	not sig	not sig
Tallman v. 132nd St	not sig	not sig	n/a, 0 v. 0

Populations Compared	Low Dose - 7 day	
	Infection Rate	Dissemination Rate
Juniper v. Aegypti	not sig	not sig
Juniper High Dose v. Juniper Low Dose	p < 0.001	p = 0.012

Table S3: Number of mosquitoes used in each experiment.

Location	Day 7 Replicate 1	Day 7 Replicate 2	Day 14 Replicate 1	Day 14 Replicate 2	Low Dose Replicate 1	Low Dose Replicate 2
Juniper	31	25	18	19	21	17
Tallman	17	23	20	19		
W 132nd	28	15	17	12		
Aegypti					26	18
